# Prefrontal-Premotor Pathways and Motor Output in Well-Recovered Stroke Patients

**DOI:** 10.3389/fneur.2019.00105

**Published:** 2019-02-14

**Authors:** Robert Schulz, Clemens G. Runge, Marlene Bönstrup, Bastian Cheng, Christian Gerloff, Götz Thomalla, Friedhelm C. Hummel

**Affiliations:** ^1^Department of Neurology, University Medical Center Hamburg-Eppendorf, Hamburg, Germany; ^2^Department of Neurology, University Medical Center Schleswig-Holstein, Lübeck, Germany; ^3^Human Cortical Physiology and Neurorehabilitation Section, National Institute of Neurological Disorders and Stroke, National Institutes of Health, Bethesda, MD, United States; ^4^Defitech Chair of Clinical Neuroengineering, Center for Neuroprosthetics and Brain Mind Institute, Swiss Federal Institute of Technology (EPFL), Geneva, Switzerland; ^5^Defitech Chair of Clinical Neuroengineering, Clinique Romande de Réadaptation, Center for Neuroprosthetics and Brain Mind Institute, Swiss Federal Institute of Technology Valais (EPFL Valais), Sion, Switzerland; ^6^Clinical Neuroscience, University of Geneva Medical School, Geneva, Switzerland

**Keywords:** diffusion, recovery, corticocortical, DLPFC, VLPFC, tractography

## Abstract

Structural brain imaging has continuously furthered our knowledge how different pathways of the human motor system contribute to residual motor output in stroke patients. Tract-related microstructure of pathways between primary and premotor areas has been found to critically influence motor output. The motor network is not restricted in connectivity to motor and premotor areas but these brain regions are densely interconnected with prefrontal regions such as the dorsolateral (DLPFC) and ventrolateral (VLPFC) prefrontal cortex. So far, the available data about the topography of such direct pathways and their microstructural properties in humans are sparse. To what extent prefrontal-premotor connections might also relate to residual motor outcome after stroke is still an open question. The present study was designed to address this issue of structural connectivity of prefrontal-premotor pathways in 26 healthy, older participants (66 ± 10 years old, 15 male) and 30 well-recovered chronic stroke patients (64 ± 10 years old, 21 males). Probabilistic tractography was used to reconstruct direct fiber tracts between DLPFC and VLPFC and three premotor areas (dorsal and ventral premotor cortex and the supplementary motor area). Direct connections between DLPFC/VLPFC and the primary motor cortex were also tested. Tract-related microstructure was estimated for each specific tract by means of fractional anisotropy and alternative diffusion metrics. These measures were compared between the groups and related to residual motor outcome in the stroke patients. Direct prefrontal-premotor trajectories were successfully traceable in both groups. Similar in gross anatomic topography, stroke patients presented only marginal microstructural alterations of these tracts, predominantly of the affected hemisphere. However, there was no clear evidence for a significant association between tract-related microstructure of prefrontal-premotor connections and residual motor functions in the present group of well-recovered stroke patients. Direct prefrontal-motor connections between DLPFC/VLPFC and the primary motor cortex could not be reconstructed in the present healthy participants and stroke patients.

## Introduction

Brain imaging has enhanced our understanding of plasticity-related functional reorganization after stroke. Within the motor domain, the focus of functional imaging based network analyses has been primarily the core motor network, comprising the primary motor cortices (M1) and secondary motor areas of the frontal lobe, such as the dorsal (PMd) and ventral (PMv) premotor cortex and the supplementary motor area (SMA). Such analyses could demonstrate that both active and passive network states and their temporal changes over time significantly relate to residual motor functioning and recovery processes (1). Diffusion-weighted imaging has shown that also the structural state of the underlying fiber tracts connecting these brain regions is associated with motor outcome. The body of literature of such structural connectivity analyses after stroke has been recently summarized (2).

Compared to the motor execution network showing prominent and clinically relevant changes in functioning and structure, much less is known about the prefrontal cortex (PFC) and its importance after ischemic stroke. Indeed, the PFC is a large brain area with multiple heterogeneous structurally and functionally defined brain regions. Studies in healthy participants have already evidenced its important role in the cognitive and higher-order motor domains including working memory (3–6). Herein, particularly the dorsolateral prefrontal cortex (DLPFC) activation has been reported in the cognitive control of task planning and learning of action sequences. Ventrolateral prefrontal cortices (VLPFC) have been found to influence emotional and visuomotor processing, action inhibition and updating of action plans, as well as object integration (3, 5). With regard to the underlying neuronal networks, animal tracing studies (7–11) and a study in healthy participants (12) have shown that DLPFC and VLPFC show various connections to other brain regions. Particularly with respect to the core motor network, both areas have been reported to show direct structural connections to multiple premotor areas; with DLPFC being primarily connected to PMd, and VLPFC to PMv. So far, neither the presence of such connections has been probed, nor have their topographical details been analyzed systematically in elderly healthy humans.

In patients after ischemic stroke, the present understanding of the contribution of PFC to motor functions is largely based on few functional imaging studies. These have reported, for instance, increased PFC activation after stroke for simple finger tapping (13), visuomotor grip tasks, particularly in more impaired patients (14), for timed hand movements (15) and during action-selection tasks (16). Motor imagery has been found to activate PFC in stroke patients (17) and to lead to enhanced excitatory coupling with PMd and SMA which might suggest a disease-specific role of cognitive related brain areas for movement preparation and planning to facilitate proper motor output (18). It has been argued that, in this way, PFC might contribute to the increasing neuronal output from the executive motor network to spinal cord motor neurons originating in premotor areas as well (14, 19). Moreover, the success of motor sequence learning after stroke has been related to PFC network activation (20, 21). This involvement in motor learning after stroke might render the PFC a potential substrate for continuous re-learning of lost motor functions during recovery (22).

Despite these data, most previous imaging studies, particularly those aiming at connectivity analyses like dynamic causal modeling (1), have largely neglected the PFC and its connections with premotor areas, often due to the lack of activation during simple motor tasks (1, 23). This is likely to continuously bias the present perception of the influence of the PFC after stroke in the motor domain besides its role in the cognitive domain (24). Similarly, a detailed analysis of the topography of prefrontal-premotor connections and their microstructural characteristics is still lacking, both in healthy participants and in stroke patients. Though, particularly such task-independent and tract-based structural analyses seem to be warranted and needed to extend our understanding of the importance of alternative brain networks (2) supporting motor outcome after stroke.

The present study was designed to address this topic of structural connectivity of prefrontal-premotor pathways. We aimed at reconstructing pathways between the DLPFC or the VLPFC and premotor areas such as PMd, PMv, and SMA, as well as the M1 by means of diffusion-based imaging and probabilistic tractography. On one hand, a group of older, healthy participants was examined to probe the presence and topography of these connections *in vivo*, and several diffusion metrics were used to quantify their microstructural properties. On the other hand, a group of well-recovered chronic stroke patients was analyzed. For this group, we hypothesized to find significant associations between the microstructural state of some of these fiber tracts and residual motor outcome. The presence or absence of such relationships might help to update priors for future studies aiming at analazing multiple motor networks simultaneously—both at the corticocortical and corticofugal level—to better understand the importance of various structural brain networks for motor recovery.

## Participants and Methods

### Subjects

**Figure 1:**
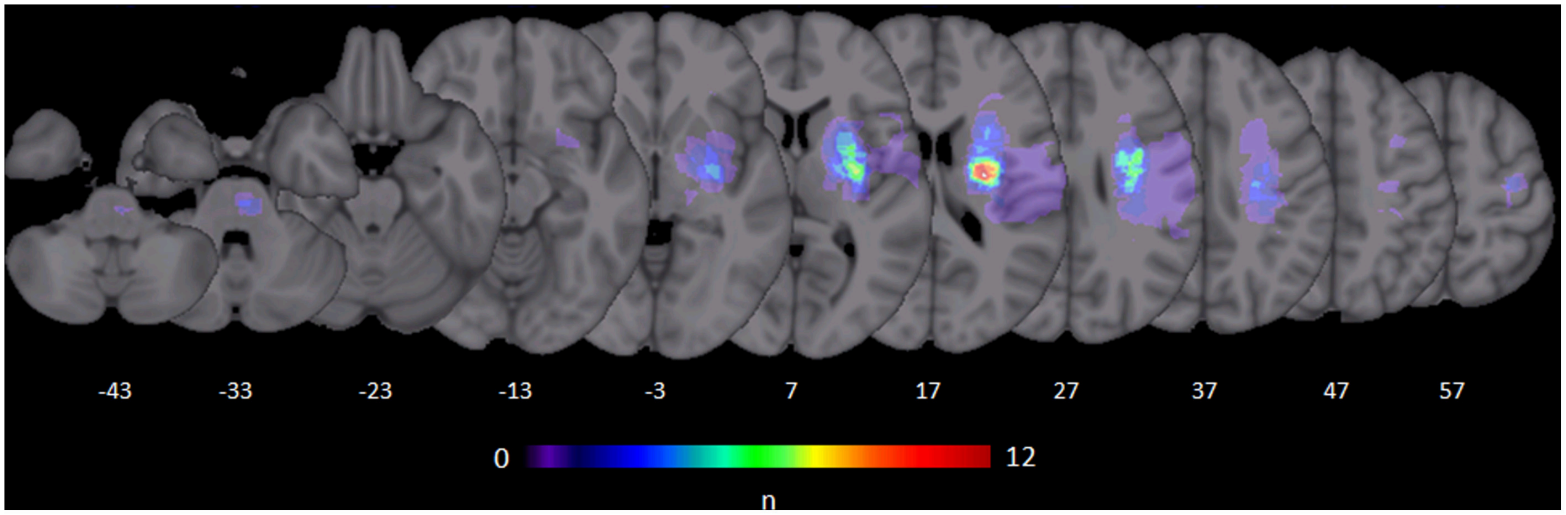
Stroke lesions. All masks of stroke lesions were brought to the right side and overlaid on a T1 template in MNI standard space. The color bar indicates the number of subjects in which voxels lay within a stroke lesion.

Thirty well-recovered patients (64.2 ± 9.7 years old (SD), median 64, range 45–82, 21 male, 3 left-handed) were included 15.5 ± 7.7 months (range 6–44) after first-ever ischemic stroke with an upper extremity motor deficit. The lesions were mainly located in subcortical areas including the brainstem. Figure 1 illustrates the distribution of stroke lesions. A subgroup of these patients (*n* = 15) has been already included in a previous study on parietofrontal structural connectivity (25).The patients were evaluated clinically by means of the Fugl-Meyer assessment of the upper extremity (UEFM) (26), a measure of motor function, that is active movement ranges and synergies of proximal and distal muscles, and grip and pinch force values (given as proportional values affected/unaffected hand, mean value over 3 consecutive measurements for each hand) (27), measures of force and residual motor output. In addition to these individual parameters, all three scores were also combined to one composite motor outcome score (MO) (27) using a factor analysis with principal component extraction (first eigenvariate accounting for 67.6% of the variance in each variable). Demographic and clinical data are summarized in Supplementary Table 1. Twenty six healthy elderly participants of similar age and sex were also analyzed (66.4 ± 9.6 years old, median 69, range 48–79, 15 male, group comparison for age and sex, n.s.). The present study was approved by the local ethics committee (PV3777). All participants gave written informed consent according to the Declaration of Helsinki.

### Brain Imaging

A 3T Siemens Skyra MRI scanner (Siemens, Erlangen, Germany) was used to acquire diffusion-weighted images as well as high-resolution T1-weighted images of the whole brain.

The diffusion-weighted images consisted of 75 axial slices with gradients (b = 1,500 s/mm^2^) applied along 64 non-collinear directions. The sequence parameters were: repetition time (TR) = 10,000 ms, echo time (TE) = 82 ms, field of view (FOV) = 256 × 204, slice thickness (ST) = 2 mm, in-plane resolution (IPR) = 2 × 2 mm. A three-dimensional magnetization-prepared, rapid acquisition gradient-echo sequence (MPRAGE) was used for high-resolution T1-weighted images. The sequence parameters were: TR = 2,500 ms, TE = 2.12 ms, FOV = 256 × 208 mm, 256 axial slices, ST = 0.94 mm and IPR = 0.83 × 0.83 mm.

### Pre-processing and Mask Creation

The FSL software package 5.1 (http://www.fmrib.ox.ac.uk/fsl) was used to analyze the diffusion-weighted and anatomical images. Prior to brain extraction, correction of eddy currents and head motion was conducted. The diffusion tensor model was fitted to each voxel and fractional anisotropy (FA) maps were calculated. FSL's *bedpostx* was used to estimate the distribution of diffusion parameters in each voxel, modeling crossing fibers using Markov Chain Monte Carlo sampling. A non-linear co-registration of the anatomical images to the individual FA maps was conducted. Both the FA maps as well as the anatomical images were then registered non-linearly to the Montreal Neurological Institute (MNI) standard space. Based on the tensor information, maps for alternative diffusion metrics that are mean diffusivity (MD), axial (AD), and radial diffusivity (RD) were also calculated. For T1 segmentation, cortical parcellation and the calculation of the cortical seed and target masks, that are SMA, PMv, PMd, M1, DLPFC, and VLPFC, we used FSL's *fast* and the Freesurfer software (http://surfer.nmr.mgh.harvard.edu/). An in-house Matlab script (Mathworks, Natick, MA, US) was used to bias the masks for SMA, PMv, PMd, and M1 toward hand representations. Details on the mask calculations are given in the Supplementary Text 1 and also previous reports (25, 27).

### Probabilistic Tractography

Structural connections were reconstructed from DLPFC and VLPFC to SMA, PMv, and PMd, respectively, applying probabilistic tractography via FSL's *probtrackx* in stroke patients and healthy participants. Also, we aimed to reconstruct probable trajectories between DLPFC and VLPFC and M1. First, 25,000 streamlines were sent from each voxel in the prefrontal seed masks VLPFC/DLPFC and also backward originating in the frontal motor masks. Both output distributions (backward/forward) (28) were then combined to estimate a tract-specific exclusion mask, in which a second tractography was conducted to control for erroneous trajectories. This procedure was already used in a previous study (25) and found to allow a reliable reconstruction of trajectories with, compared to others, small structural connectivity probabilities. The final probabilistic tractography distribution (28) was then analyzed applying four different thresholds from 1 to 10% (from more liberal to more restricted spatial extent). Tract-related mean FA, a widely used diffusion metric and surrogate parameter of white matter microstructure, was calculated for each tract and averaged across all four thresholds. Details on this procedure are given in Supplementary Text 2. Tract-related alternative diffusions metrics MD, AD, and RD values were also estimated to provide a more detailed picture about the microstructural characteristics of the prefrontal-premotor connections. Herein, as inverse measures of membrane density, MD and RD increases have been reported to parallel white matter demyelination, whereas AD decreases have been primarily correlated with axonal injury (29). Data of 26 healthy participants were also analyzed to allow for group comparisons. To account for the distribution of dominant and non-dominant hemispheres affected, the right and left hemispheres of the healthy participants were pseudo-randomly assigned to the “affected” (AH, right) and “unaffected” (UH, left) hemisphere, respectively. To assess the topographic distribution of each tract, we applied a center-of-gravity (COG) analysis along coronal slices in MNI space in steps of 2 mm from y = −40 to y = 70. Tract-related COG coordinate values were calculated for each participant, tract and threshold, and averaged across all thresholds in each slices. Only coordinates comprising values of at least two of the thresholds were considered.

As the corticospinal tract (CST) from M1 critically influences residual motor outcome after stroke (19), structure-function relationships for the corticocortical connections were analyzed by accounting for the integrity of the CST. Templates for this tract, originating from M1 hand area, derived from 26 healthy participants, were available for both sides from a previous study (27). Using these templates, tract-related FA values for the CST were calculated for the affected and unaffected hemispheres at the level from the mesencephalon to the cerebral peduncle (MNI: z = −25 to z = −20), given as proportional values affected/unaffected hemispheres.

### Statistics

R (version 3.3.2) and RStudio (version 1.0.136) were used for the statistical analyses. R's *lmer* function for linear mixed-effects modeling with repeated measures was used to compare tract-related diffusion metrics (separate models for FA, MD, RD, AD values, each value as the dependent variable) for every tract of interest (effect TRACT) of both hemispheres (effect SIDE) between stroke patients and healthy participants (effect GROUP). Stepwise back elimination of relevant non-significant interactions (GROUP^*^TRACT^*^SIDE, GROUP^*^TRACT, GROUP^*^SIDE) and main effects was conducted for model simplification. The effects of age (AGE) and whether the dominant or non-dominant hemisphere was affected by the stroke (effect DOM) were included in the models as covariates. For group comparisons of COG coordinates of the tract locations, we used individual Student's *t*-tests (unpaired, 2-tailed). To explore structure-function relationships, we used R's *lm* function for multiple linear regression modeling. Separate models were fitted for each diffusion metric (FA, MD, RD, and AD) and with grip force, pinch force, UEFM, or MO, respectively, as the dependent variables. Here, AGE, DOM and also time after stroke (effect TAS) were included as covariates. Additionally, the level of damage to the CST was included in the models to account for its influence on motor outcome in chronic stroke patients (27). *Post-hoc*, we evaluated separate, additional models with lesion size (*log*-transformed) as another covariate to investigate its influence on structure-function relationships. Results are given as mean ± standard deviation (SD) or 95% confidence intervals (CI) as indicated. Statistical significance was assumed at *P* < 0.05 corrected by means of FDR correction (30), *P*-values were also reported as uncorrected values. The level of significance was indicated by asterisks, ^*^P < 0.05, ^**^P < 0.01, uncorrected.

## Results

### Probabilistic Tractography of Prefrontal-Premotor Pathways

**Figure 2:**
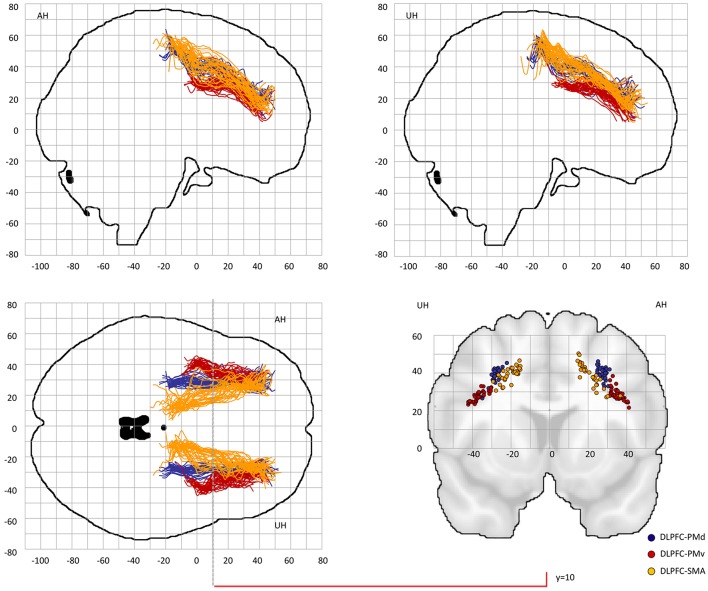
Center-of-gravity analysis of prefrontal-premotor connections in chronic stroke patients (DLPFC). The mean center-of-gravity coordinate of all given tracts and patients was calculated from y = −40 to y = 70 (MNI standard space) in 2 mm steps. Notably, only those y-values were presented in which more than two thresholds contributed to the final coordinate. All individual tracts are shown on two sagital slices, one horizontal slice and one coronal slice at y = 10. Table 1 provides statistics on the center-of-gravity analysis at the coronal level. DLPFC, dorsolateral prefrontal cortex; PMd, dorsal premotor cortex; PMv, ventral premotor cortex; SMA, supplementary motor area.

**Figure 3:**
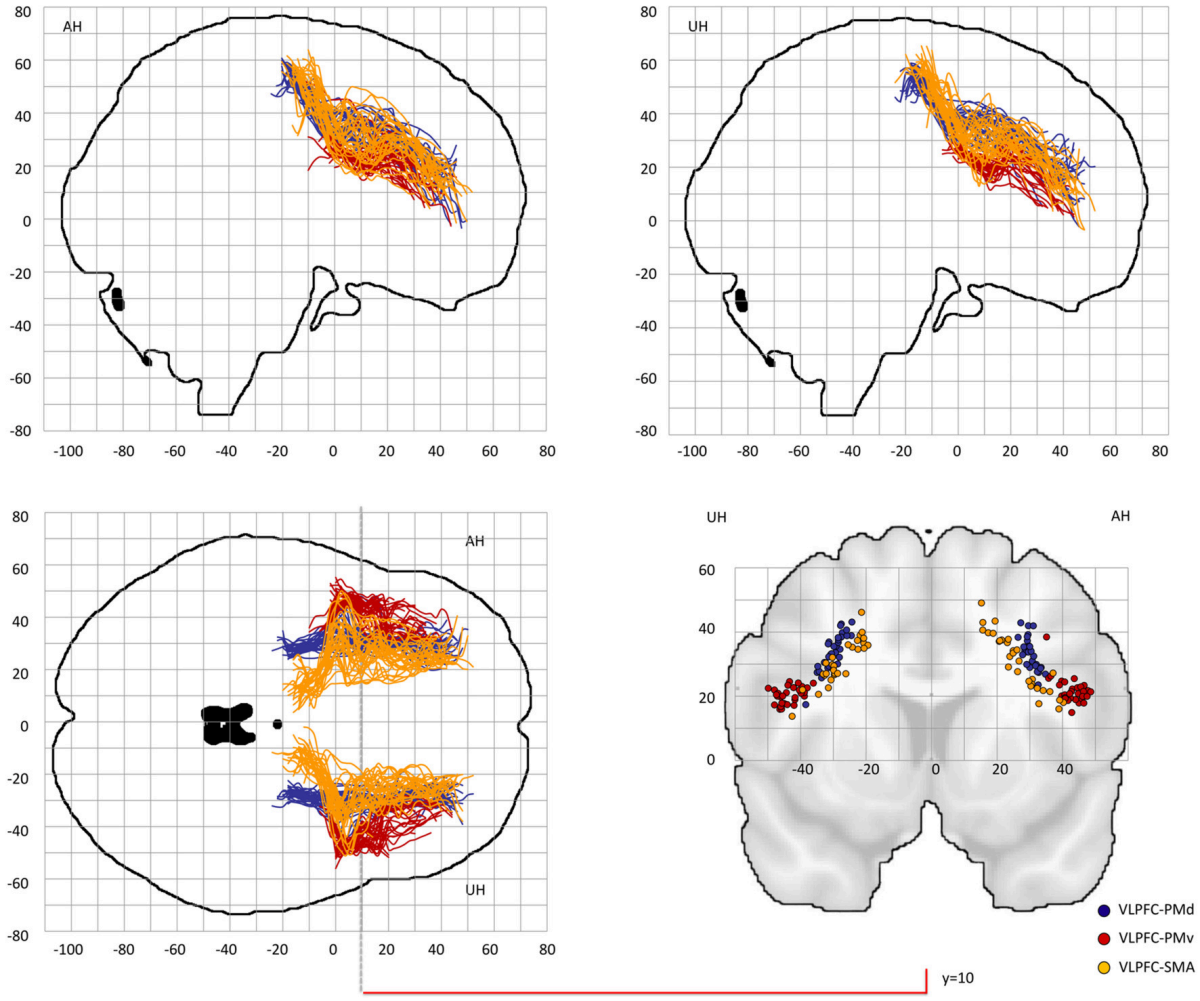
Center-of-gravity analysis of prefrontal-premotor connections in chronic stroke patients (VLPFC). The mean center-of-gravity of all given tracts and patients was calculated from y = −40 to y = 70 (MNI standard space) in 2 mm steps. Notably, only those y-values were presented in which more than two thresholds contributed to the final coordinate. All individual tracts are shown on two sagital slices, one horizontal slice and one coronal slice at y = 10. Table 1 provides statistics on the center-of-gravity analysis at the coronal level. VLPFC, ventrolateral prefrontal cortex; PMd, dorsal premotor cortex; PMv, ventral premotor cortex; SMA, supplementary motor area.

Probable trajectories connecting DLPFC and VLPFC with PMv, PMd, and SMA were reconstructed in the stroke patients and healthy participants. For PMv- and PMd-related connections we found similar spatial distributions across participants. As indicated by the center-of-gravity topographic analysis for DLPFC-derived tracts in Figure 2 and VLPFC in Figure 3 (see also Table 1 and Supplementary Figures 1, 2 for the results of the healthy participants), the majority of prefrontal-premotor pathways were located in the 2nd component of the superior longitudinal fascicle (SLF II). Trajectories connecting VLPFC and PMv appeared to be located rather in the 3rd than 2nd component (SLF III). With high spatial variability particularly in the stroke patients, SMA-related trajectories to DLPFC and VLPFC were partly located also in the 1st part of the superior longitudinal fascicle (SLF I) (25, 31, 32). Compared to the healthy participants, the patients showed a more laterocaudal mean distribution of DLPFC-SMA fibers in the unaffected hemisphere (Table 1). All other tracts did not show significant group differences in this coronal position. Overall, there was an anatomically plausible topographic distribution of prefrontal connections targeting SMA, PMv, and PMd with the connection of PMv to DLPFC/VLPFC being located in a ventrolateral position and DLPFC/VLPFC-PMd being located more medially. We also sought to reconstruct probable connections between DLPFC/VLPFC and M1. However, this did not result in successful tracking. In most cases the trajectories reconstructed by our approach included pathways through the primary sensory cortex (S1, postcentral gyrus). Hence, it was not possible to isolate direct connections from DLPFC/VLPFC to M1 from potential indirect connections via S1. Consequently, the connection between M1 and DLPFC/VLPFC was excluded from further analyses (data not shown).

**Table 1:** Tract-related centers-of-gravity of DLPFC/VLPFC connections at y = 10 (MNI) for stroke patients and healthy participants.

	**Group**	**DLPFC-PMd**		**DLPFC-PMv**		**DLPFC-SMA**	
		**AH**	**UH**	**AH**	**UH**	**AH**	**UH**
X	Stroke	28.03 (±2.16)	−28.33 (±2.04)	35.22 (±3.12)	−37.18 (±3.40)	20.77 (±5.48)	−20.69^**^ (±4.14)
	Control	27.43 (±1.68)	−27.64 (±2.91)	36.91 (±3.33)	−36.74 (±2.88)	18.99 (± 4.24)	−17.68 (±2.79)
Z	Stroke	39.73 (±3.58)	38.58 (±3.84)	28.60 (±3.53)	27.71 (±2.78)	39.46 (±6.13)	39.02^**^ (±3.88)
	Control	40.29 (±2.38)	39.60 (±3.66)	27.89 (±2.68)	28.58 (±2.50)	41.63 (±3.70)	42.23 (±2.98)
	**Group**	**VLPFC-PMd**		**VLPFC-PMv**		**VLPFC-SMA**	
		**AH**	**UH**	**AH**	**UH**	**AH**	**UH**
x	Stroke	30.26 (±1.94)	−29.91 (±3.25)	42.86 (±3.55)	−43.45 (±3.50)	26.65 (±7.40)	−28.08 (±5.86)
	Control	29.92 (±1.82)	−30.79 (±3.12)	43.62 (±3.02)	−43.33 (±2.51)	29.22 (± 6.74)	−28.54 (±5.27)
z	Stroke	33.51 (±4.83)	34.20 (±5.74)	21.77 (±4.08)	20.36 (±2.50)	30.80 (±8.86)	30.49 (±6.85)
	Control	33.58 (±4.18)	32.31 (±6.79)	20.07 (±2.74)	20.65 (±2.18)	28.09 (±8.07)	28.59 (±6.33)

*Centers-of-gravity of prefrontal-premotor connections at the mid-level of the connections (y = 10) for stroke patients and healthy participants. Individual x- and z-values are given as mean (±SD) for the affected (AH) and unaffected (UH) hemisphere for both groups. DLPFC, dorsolateral prefrontal cortex; VLPFC, ventrolateral prefrontal cortex; PMd, dorsal premotor cortex; PMv, ventral premotor cortex; SMA, supplementary motor area. Significant group differences are indicated by asterisks*.

### Tract-Related White Matter Microstructure of Prefrontal-Premotor Connections

Linear mixed-effects models with repeated measures were estimated to compare tract-related microstructure between stroke patients and healthy participants. For tract-related FA, we did not find tract- and side-specific group differences (GROUP^*^TRACT^*^SIDE interaction: *F* = 1.58, *P* = 0.17). However, the simplified model showed a significant GROUP effect (*F* = 5.54, *P* = 0.02), indicating an unspecific, only marginal reduction of prefrontal-premotor FA in chronic stroke patients with estimated FA mean values [95% CI] of 0.33 [0.32–0.34] for patients and 0.34 [0.34–0.35] for healthy participants). Table 2 summarizes tract-related mean FA values for both hemispheres and both groups. Triple interaction was similarly not significantly contributing to the models for the other diffusion metrics MD (*F* = 1.75, *P* = 0.15), RD (*F* = 2.02, *P* = 0.10), and AD (*F* = 0.86, *P* = 0.51). For MD however, there was a significant GROUP^*^SIDE interaction (*F* = 13.80, *P* < 0.01) with only marginally increased tract-related MD in the tracts on the affected hemisphere in stroke patients compared to the healthy participants. Likewise, also RD values (GROUP^*^SIDE, *F* = 8.34, *P* < 0.01) and AD values were slightly higher in the lesioned hemisphere of the patients (GROUP^*^SIDE interaction: *F* = 20.50, *P* < 0.01). MD, RD, and AD mean values are given in Supplementary Table 3.

**Table 2:** Tract-related white matter microstructure in stroke patients and healthy participants.

		**DLPFC-PMd**		**DLPFC-PMv**		**DLPFC-SMA**	
		**AH**	**UH**	**AH**	**UH**	**AH**	**UH**
FA	Stroke	0.34 (±0.03)	0.35 (±0.02)	0.31 (±0.03)	0.32 (±0.03)	0.36 (±0.03)	0.36 (±0.03)
	Control	0.35 (±0.03)	0.35 (±0.02)	0.32 (±0.03)	0.33 (±0.03)	0.37 (±0.03)	0.38 (±0.04)
		**VLPFC-PMd**		**VLPFC-PMv**		**VLPFC-SMA**	
		**AH**	**UH**	**AH**	**UH**	**AH**	**UH**
FA	Stroke	0.34 (±0.03)	0.35 (±0.02)	0.26 (±0.03)	0.26 (±0.02)	0.36 (±0.03)	0.37 (±0.03)
	Control	0.36 (±0.03)	0.36 (±0.02)	0.26 (±0.03)	0.26 (±0.03)	0.38 (±0.02)	0.38 (±0.03)

*Overview of mean FA values presented as means (±SD) for the affected (AH) and unaffected (UH) hemisphere. FA, fractional anisotropy; DLPFC, dorsolateral prefrontal cortex; VLPFC, ventrolateral prefrontal cortex; PMd, dorsal premotor cortex; PMv, ventral premotor cortex; SMA, supplementary motor area*.

### Tract-Related White Matter Microstructure and Residual Motor Outcome After Stroke

Individual multiple linear regression models were fitted for tract-related FA values for all prefrontal-premotor connections to estimate their influence on aspects of residual motor output in chronic stroke patients. Table 3 summarizes the estimated coefficients for each tract with uncorrected *P*-values following the exploratory approach of the present study. Of note, after *post-hoc* false-discovery-rate (FDR) correction for 48 tests (30), there was no significant association for any of the tracts of interest, neither for residual motor output (grip, pinch forces), motor functions (UEFM) nor gross motor outcome (MO). Without correction though, we observed a significant positive influence of tract-related FA and pinch force (*P* < 0.01) for the connection DLPFC-PMv of the unaffected hemisphere. However, for whole-hand grip force and UEFM, the same tract did not show a similar structure-function association (*P* = 0.46 and *P* = 0.47, respectively). In order to explore the nature of this relationship in more detail, we also analyzed tract-related MD, RD and AD values. For DLPFC-PMv of the unaffected hemisphere, we found negative correlations with pinch force values for MD and RD values (*P* < 0.01), tract-related AD was not related to pinch forces. Similarly, all other behavioral measures were not related to MD, RD, or AD values of this specific tract. Finally, there was a negative correlation between UEFM and tract-related FA for DLPFC-PMv (*P* = 0.045) for the affected and a positive correlation between UEFM and tract-related AD for VLPFC-PMv (*P* = 0.03) of the unaffected hemisphere. All other models did not show significant results (see Supplementary Tables 4–6 for estimated coefficients for tract-related MD, RD and AD values). *Post-hoc*, we explored whether lesion sizes (*log*-transformed) would influence these findings. However, including this additional covariate in the models did not change the present modeling results (see Supplementary Tables 7–10).

**Table 3:** Tract-related mean FA and residual motor output after stroke.

**Parameter**	**Side**	**Tract**	**Estimated coefficient**	**Confidence interval**		***T*-Value**	***P*-Value**
				**Lower**	**Upper**		
Grip	AH	DLPFC-PMd	0.35	−1.49	2.20	0.40	0.70
		DLPFC-PMv	−1.02	−2.92	0.89	−1.10	0.28
		DLPFC-SMA	0.07	−1.59	1.72	0.08	0.93
	UH	DLPFC-PMd	0.70	−1.71	3.10	0.60	0.56
		DLPFC-PMv	0.70	−1.22	2.63	0.76	0.46
		DLPFC-SMA	0.56	−1.46	2.57	0.57	0.57
	AH	VLPFC-PMd	0.05	−1.99	2.09	0.05	0.96
		VLPFC-PMv	0.03	−2.50	2.57	0.03	0.98
		VLPFC-SMA	−0.06	−1.83	1.72	−0.06	0.95
	UH	VLPFC-PMd	1.01	−1.46	3.49	0.85	0.41
		VLPFC-PMv	0.96	−2.56	4.47	0.56	0.58
		VLPFC-SMA	0.02	−1.97	2.01	0.02	0.99
Pinch	AH	DLPFC-PMd	1.72	−0.54	3.99	1.57	0.13
		DLPFC-PMv	0.66	−1.84	3.16	0.54	0.59
		DLPFC-SMA	0.56	−1.55	2.67	0.55	0.59
	UH	DLPFC-PMd	0.78	−2.32	3.88	0.52	0.61
		DLPFC-PMv	3.48	1.46	5.51	3.55	<0.01^**^
		DLPFC-SMA	0.77	−1.82	3.35	0.61	0.55
	AH	VLPFC-PMd	2.29	−0.16	4.73	1.93	0.07
		VLPFC-PMv	0.53	−2.72	3.78	0.34	0.74
		VLPFC-SMA	0.89	−1.35	3.14	0.82	0.42
	UH	VLPFC-PMd	1.31	−1.87	4.48	0.85	0.41
		VLPFC-PMv	−0.77	−5.31	3.77	−0.35	0.73
		VLPFC-SMA	0.77	−1.76	3.31	0.63	0.53
UEFM	AH	DLPFC-PMd	−16.26	−63.79	31.26	−0.71	0.49
		DLPFC-PMv	−47.72	−94.34	−1.11	−2.11	0.045^*^
		DLPFC-SMA	−5.05	−47.95	37.84	−0.24	0.81
	UH	DLPFC-PMd	−11.05	−73.81	51.71	−0.36	0.72
		DLPFC-PMv	17.87	−32.09	67.82	0.74	0.47
		DLPFC-SMA	4.26	−48.41	56.92	0.17	0.87
	AH	VLPFC-PMd	−23.10	−75.24	29.04	−0.91	0.37
		VLPFC-PMv	−12.54	−78.19	53.11	−0.39	0.70
		VLPFC-SMA	−20.08	−65.27	25.10	−0.92	0.37
	UH	VLPFC-PMd	−12.48	−77.42	52.47	−0.40	0.70
		VLPFC-PMv	−10.95	−102.79	80.89	−0.25	0.81
		VLPFC-SMA	−9.52	−60.99	41.96	−0.38	0.71
MO	AH	DLPFC-PMd	4.37	−12.42	21.17	0.54	0.60
		DLPFC-PMv	−8.35	−25.86	9.16	−0.98	0.33
		DLPFC-SMA	1.29	−13.81	16.39	0.18	0.86
	UH	DLPFC-PMd	3.33	−18.77	25.42	0.31	0.76
		DLPFC-PMv	16.10	−0.32	32.53	2.02	0.05
		DLPFC-SMA	5.01	−13.41	23.43	0.56	0.58
	AH	VLPFC-PMd	4.11	−14.47	22.69	0.46	0.65
		VLPFC-PMv	0.00	−23.17	23.17	0.00	1.00
		VLPFC-SMA	−0.26	−16.43	15.92	−0.03	0.97
	UH	VLPFC–PMd	5.92	−16.87	28.70	0.54	0.60
		VLPFC-PMv	−0.65	−33.00	31.70	−0.04	0.97
		VLPFC-SMA	1.15	−17.01	19.31	0.13	0.90

*Individual multiple linear regression models were estimated for each tract to test the relationship between tract-related mean FA values and the four behavioral parameter grip force (Grip), pinch force (Pinch) values, UEFM score, and the composite score MO. Estimated coefficients of the tract-related FA values were adjusted for age, lesioned hemisphere (dominant or non-dominant), time after stroke and CST integrity and are given with 95% confidence intervals. P-values are derived from each model separately and were not corrected for multiple testing. Note that after correction for 48 models (30), none of the tracts reached the level of significance. Estimated coefficients of the four covariates are not shown for the sake of clarity. AH, affected hemisphere, UH, unaffected hemisphere. Asterisks indicate significant tracts*.

## Discussion

In the present study, we determined the topography and microstructural state of prefrontal-premotor connections of the human brain in a group of healthy aged participants and well-recovered chronic stroke patients. The data show that prefrontal-premotor trajectories are traceable in both groups. Similar in gross anatomic topography, stroke patients presented only marginal microstructural alterations of these tracts, predominantly of the affected hemisphere. However, there was no clear evidence for a significant association between tract-related microstructure of prefrontal-premotor connections and residual motor functions after stroke.

Using probabilistic tractography, we were able to reconstruct probable trajectories connecting DLPFC and VLPFC with PMd, PMv, and SMA in older healthy participants and chronic stroke patients. For DLPFC, this was in good agreement with tracing data in monkeys reporting strong connections between DLPFC and the whole extent of PMv equivalent areas. For SMA and PMd, DLPFC-premotor trajectories have been found to be restricted only to premotor regions, which are connected to M1, with higher connectivity for SMA than for PMd (7). Other tracing studies have reported variable connection strengths for DLPFC-PMd (8–10). Previous tractography data in humans have shown comparable connection strengths for DLPFC-PMv and DLPFC-PMd (12). For VLPFC, pathways have been traced in monkeys to PMv and, to a lesser extent, also to PMd equivalent brain regions (8, 10, 11). Similar findings have been reported for humans (12). Hence, the available data for direct DLPFC/VLPFC-SMA connections in monkeys seem to be rather inconclusive. Given increased spatial variability of these connections also in our sample, the allocation of prefrontal-SMA trajectories to SLF I remains relatively vague and should be interpreted with caution. As these trajectories were found to cross from the lateral to the medial surface of the frontal lobe, other crossing fibers of the SLF are likely to influence the present tract reconstructions. More elaborated diffusion-based imaging techniques, e.g., based on constrained spherical deconvolution (33), might be helpful and needed to verify the present results. Nevertheless, the present study provides first topographic connectivity data for prefrontal-premotor pathways in healthy humans and also stroke patients *in vivo*. One study has already reported connection strengths for PMv and PMd in healthy participants, but has not investigated the topography of the underlying pathways (12). Here, PMv- and PMd-related connections of DLPFC and VLPFC could be largely allocated to SLF II and VLPFC-PMv trajectories to SLF III. In fact, these distributions are well in line with previous data in monkeys (34). Finally, for M1, our data corroborated previous monkey studies arguing against the existence of direct prefrontal-primary motor connection (7, 10, 35–37) indicating that prefrontal cortices are likely to influence the core motor network indirectly via premotor regions and not directly via M1.

We assessed tract-related microstructure by means of different diffusion metrics with a primary focus on tract-related mean FA as a surrogate parameter of white matter integrity, a complex, indirect measure influenced by axonal diameter and fiber density, coherence of fiber bundles and other biophysical properties (38). In order to draw a more complete picture, alternative diffusion metrics MD, RD and AD, were also assessed. We found an unspecific and only marginal reduction of tract-related FA of all tracts in the stroke patients with slightly increased tract-related MD, RD also AD values of the pathways of the affected hemisphere compared to that of the healthy participants. Previous whole brain analyses have revealed white matter changes in prefrontal-premotor brain regions in subacute (39) but not in chronic stroke patients (40, 41). The interpretation of these results (39) is neither simple nor straightforward due to technical limitations. The association fibers of the SLF investigated are crossed by corticofugal fibers, e.g., originating from the premotor areas. Thus, single voxels are likely to include multiple fiber populations with variable orientations. The validity of single-tensor derived measures is likely to be limited.

With regard to structure-function relationships, the present explorations of prefrontal-premotor and prefrontal-motor tracts did not reveal a relevant association between the microstructural properties of these tracts and residual motor outcome after stroke. There are some factors that might serve as potential explanations. First, the present sample included largely very well-recovered stroke patients in the chronic stage of recovery. In fact, previous task-related functional imaging studies have reported increased bilateral prefrontal brain activation for simple hand movements in severely impaired patients early after stroke (42) and in patients with variable deficits and intervals after stroke (41). Another study has found a negative correlation between bilateral DLPFC activation and CST integrity (14). In terms of network analyses, there is only very limited data on potential interactions of DLPFC with other brain regions and their relevance for motor output after stroke, both for task-dependent (18) and task-free resting-state analyses (43, 44). For instance, Park et al. have found in more impaired patients (mean UEFM at onset = 24) that early functional connectivity between ipsilesional M1 and contralesional DLPFC relates to motor output 6 months later (43). Hence, the present negative result overall might be explained by the high level of recovery and the time point after stroke and might change in more severely impaired patients in the acute or subacute phase after stroke. A future longitudinal study across various stages of recovery might add to a more detailed understanding of the importance of prefrontal-premotor connections for stroke recovery. Second, clinically relevant measures of residual motor output (grip and pinch forces) and motor function (UEFM) and their statistical combination via factor analysis were used to examine structure-behavior relationships in the stroke patients. Hence, the cognitive load of these tasks might have been too small for the present well-recovered stroke patients to uncover significant associations between prefrontal-premotor connections and motor function.

Some limitations of the present analysis are further worth to consider. First, patients with mainly subcortical lesions sparing prefrontal brain regions were included. To what extent our findings for prefrontal-premotor connections—remote from the lesion—can be generalized to patients with lesions directly affecting these connections remains a topic for future studies. Second, rather simple behavioral measures were used for structure-function analyses. To what extent structural properties of prefrontal-premotor connections might contribute to more complex functions (24), motor output and learning processes after stroke also remains to be investigated by future studies. For instance, cognitively more demanding tasks such as dual tasks might be able to uncover a functional importance of prefrontal-premotor structural connectivity in stroke patients. Third, based on clear a priori hypotheses, we focused on defined prefrontal-premotor connections at the cortical level. The present results are not exhaustive, and the state of other motor networks, such as prefrontal-subcortical (45) or prefrontal-cerebellar circuits, might also influence comparable analyses.

## Author Contributions

RS: study idea, study design, data acquisition, data analysis, interpretation, preparation of manuscript. CR: data analyses, interpretation, preparation of manuscript. MB and BC: data acquisition, revision of manuscript. GT and CG: providing data, interpretation, revision of manuscript. FH: study idea, study design, providing data, interpretation, and revision of manuscript.

### Conflict of Interest Statement

The authors declare that the research was conducted in the absence of any commercial or financial relationships that could be construed as a potential conflict of interest.
