# Supplementary material for: Prefrontal-Premotor Pathways and Motor Output in Well-Recovered Stroke Patients
Source: Front Neurol. 2019 Feb 14;10:105. doi: 10.3389/fneur.2019.00105 (PMC6382735; doi:10.3389/fneur.2019.00105)
Supplement: Supplementary file 1 [file Data_Sheet_1.pdf]

Supplementary Material
Prefrontal-Premotor Pathways and Motor Output in Well-Recovered Stroke Patients

Robert Schulz MD, Clemens Runge MD, Marlene Bönstrup MD, Bastian Cheng MD, Christian Gerloff MD, Götz Thomalla MD and Friedhelm C. Hummel MD

1 Supplementary Text

Text 1 | Mask calculation

To calculate cortical masks for the seed and target regions for the tractography, the T1 structural image was used for brain segmentation into white matter (WM) and grey matter (GM). The Freesurfer image analysis suite (http://surfer.nmr.mgh.harvard.edu/) was used for subsequent automatic cortical parcellation. The procedural details are given elsewhere (1). For M1, PMd and PMv, a surface-based cortical grey matter mask for the precentral gyrus was calculated based on the freesurfer parcellation labels *1024/2024* (left/right hemisphere). For SMA, we considered the superior frontal gyrus (labels 1028/2028). These large masks were transferred to FSL's MNI-T1 1mm standard space (by FLIRT/FNIRT registration) and used to calculate individual smaller masks for each motor area related to the white matter and grey matter boundary. For this, we considered both approved anatomical and connectivity based suggestions for the parcellation for whole M1 (2), SMA-proper (3) and PMd and PMv(4), as well as borders suggested by functional (5) and probabilistic cytoarchitectonic maps (6) in determining the extent of each cortical motor mask. For DLPFC, we decided to use Brodmann area (BA) 46 that is approximated as the middle third of medial frontal gyrus with the adjacent rostral portion of the inferior frontal gyrus and the middle frontal sulcus (7) (freesurfer labels *1109/2109*, *1154/2154* and *1155/2155*). The same extent was used in a previous analysis (8). For VLPFC, BA 44, 45 and 47 with the respective freesurfer labels *1018/2018*, *1019/2019* and *1020/2020* were considered. The GM/WM boundary mask was calculated using FSL-FAST segmenting the individual T1 image into one GM and one WM binary mask. These masks were combined by thresholding each mask at 0.2, binarized, mean dilatated and finally multiplied with each other. The broad masks derived from Freesurfer were multiplied with this border mask and finally binarized. Then, all cortical masks were further multiplied with binarized and normalized FA maps with a threshold at>0.1 to provide seed/target voxels with a reasonable connection to major white matter trajectories. For M1, PMd, PMv and SMA we additionally included functional imaging data into the mask creation in order to bias the reconstruction of corticocortical connections toward the hand representations within each cortical motor area. Therefore, we used average peak coordinates from previously published functional MRI data (5) and selected the 500 nearest voxels adjacent to these coordinates within each GM/WM boundary masks (using an in-house Matlab script, Matlab R2010b, Mathworks, US, see supplementary table 2) resulting in smaller motor masks related to hand function and standardized in size and relation to the cortical GM/WM boundary. This information is adapted from our previous reports(8,9). A visualization of different steps of the mask creation can be found in supplementary Fig. 2 of Schulz et al., 2015.(8)An illustrative example of final individual seed/target masks is given in Fig. 3 below.

Text 2 | Probabilistic tractography

For tractography, 25.000 streamlines were sent bi-directionally from both the seed and target regions. Interhemispheric und subcortical exclusion masks were used to guide this first step of the tract reconstruction. After equally combining both directions into one tract (10) and applying a threshold of 2%, the resulting trajectory was mean dilated four times including the seed and target masks. Based on

a refined exclusion mask derived from this procedure, to which additional exclusion voxels were added to avoid erroneous and spurious trajectories, such as corticofugal projections, a second tractography was conducted from both masks. According to a previous study (11), this procedure was found to allow for valid tract reconstruction of trajectories with - compared to others - small structural connectivity probabilities. Again, the connectivity distributions of both directions were combined. Four different levels (1%, 2%, 5% and 10%) were applied to the final output. For each threshold, the tract-related diffusion metrics were estimated and averaged across the thresholds for both the affected and unaffected hemispheres.

2 Supplementary Figures and Tables

ID	Age	Sex	Side	Dom	TAS	Grip	Pinch	UEFM	MO
1	58	F	R	0	20	0.89	1.15	64	0.90
2	57	M	L	1	20	0.75	0.80	60	-1.29
3	71	M	R	0	27	0.66	0.82	49	-3.12
4	62	M	L	1	28	0.95	1.02	63	0.57
5	63	M	L	1	12	0.79	0.84	66	-0.15
6	58	M	L	1	26	1.03	0.82	59	-0.36
7	65	M	L	1	12	0.91	1.13	66	1.21
8	69	M	R	1	11	0.89	0.88	66	0.35
9	69	F	R	0	12	1.06	0.79	66	0.64
10	71	M	R	0	12	0.99	1.06	66	1.24
11	73	F	R	0	12	0.90	0.90	66	0.42
12	66	M	R	0	14	0.77	1.17	64	0.53
13	52	M	L	1	19	0.89	1.20	66	1.36
14	66	F	R	1	23	0.63	0.80	55	-2.43
15	49	M	L	1	11	1.02	1.05	66	1.33
16	82	F	R	0	12	1.00	0.62	66	-0.10
17	56	F	R	0	13	0.91	1.04	66	0.90
18	72	F	L	1	12	1.04	1.16	65	1.60
19	64	M	L	1	12	0.89	0.96	60	-0.31
20	64	M	R	0	13	0.42	0.82	60	-2.38
21	59	M	L	1	44	0.82	0.77	59	-1.25
22	59	M	R	0	15	0.53	0.75	58	-2.51
23	70	M	R	0	13	0.87	1.19	66	1.22
24	54	M	R	0	11	0.79	0.89	66	0.00
25	79	F	L	1	12	0.97	1.04	66	1.13
26	45	M	L	0	11	0.82	1.00	66	0.48
27	62	M	R	0	6	0.64	0.74	59	-2.00
28	49	M	R	0	8	0.63	0.90	60	-1.39
29	82	M	L	1	12	1.03	0.97	66	1.09
30	79	F	L	1	12	1.10	1.28	66	2.32

Tab. 1 | Clinical characteristics

M male, F female. R right, L left. Dom indicates whether the dominant hemisphere was affected (1) or not affected (0). TAS Time after stroke in months. Grip/Pinch indicate the proportional values of the ratio affected/unaffected whole hand grip force (kg) or pinch force (kg), respectively. UEFM Fugl-Meyer score of the upper extremity. MO = composite motor output score for gross motor outcome and based on grip, pinch and UEFM.

	Right			Left		
	x	y	z	x	y	z
**M1**	37	-25	62	-37	-25	62
**PMd**	30	-7	63	-30	-7	63
**PMv**	51	4	24	-51	4	24
**SMA**	2	-10	59	-2	-10	59

*Tab. 2 |MNI coordinates of peak activation within cortical motor sites [Tailarach-MNI converted] (5,12)*Note that for DLPFC and VLPFC the whole extent of the mask was used during probabilistic tractography.

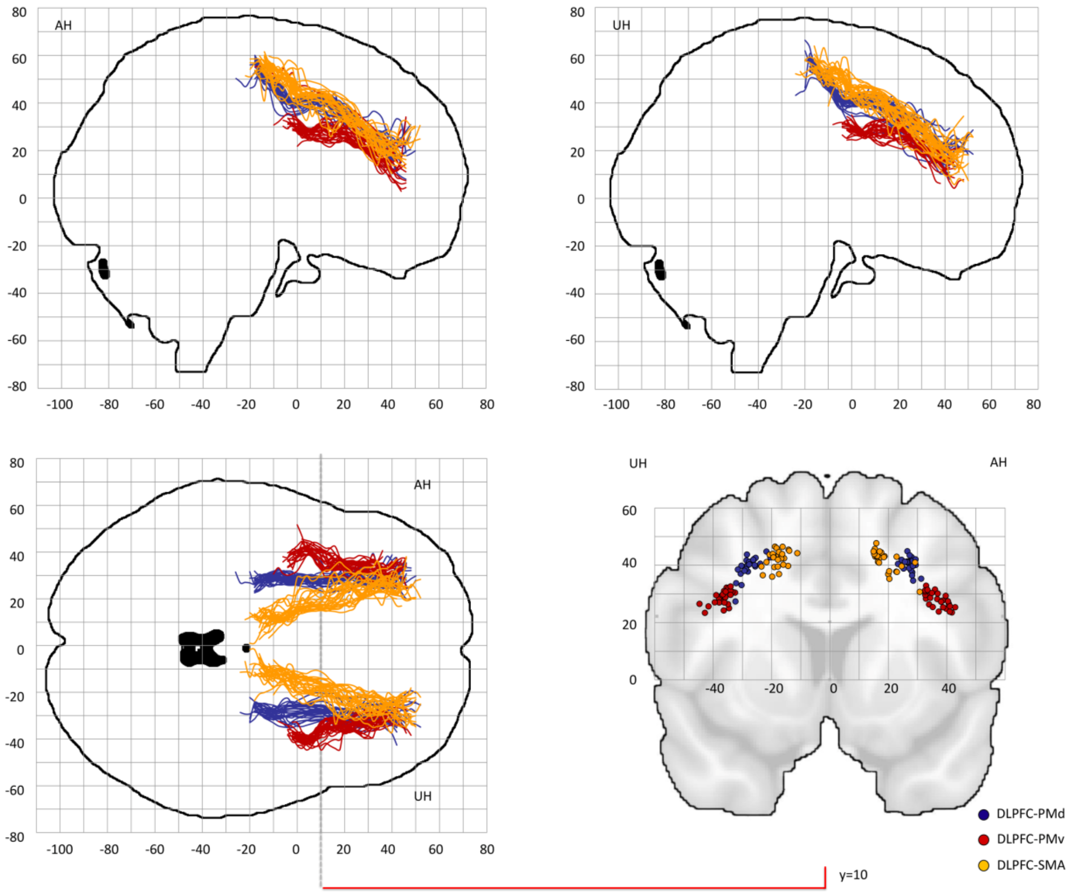

Fig. 1 | Centre of gravity analysis of prefrontal-premotor connections in healthy controls (DLPFC) The mean centre of gravity of all given tracts and patients was calculated from y=-40 to y=70 (MNI standard space) in 2 mm steps. Notably, only those y-values were presented in which more than two thresholds contributed to the final coordinate. All individual tracts are shown on two sagital slices, one horizontal slice and one coronar slice at y=10. Table 2 provides statistics on the centre of gravity analysis at the coronar level. DLPFC = dorsolateral prefrontal cortex; PMd = dorsal premotor cortex; PMv = ventral premotor cortex; SMA = supplementary motor area.

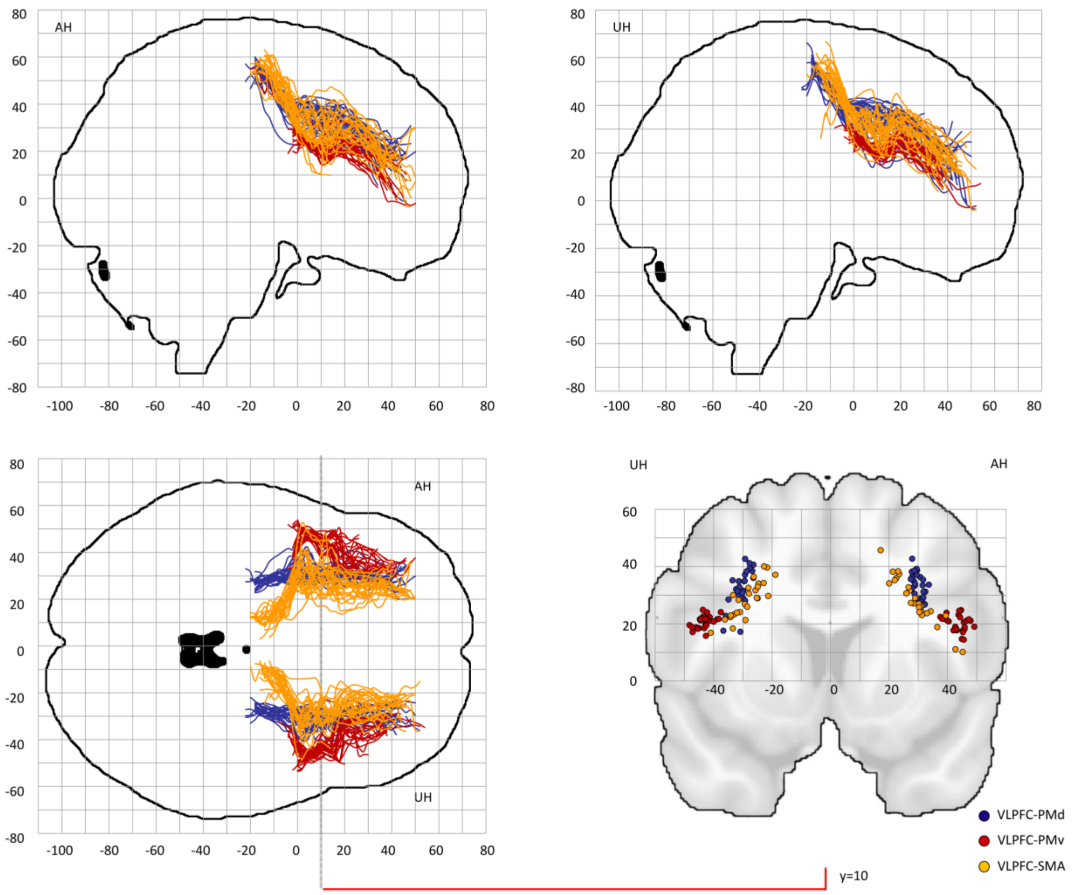

Fig. 2 | Centre of gravity analysis of prefrontal-premotor connections in healthy controls (VLPFC)
The mean centre of gravity of all given tracts and patients was calculated from y=-40 to y=70 (MNI standard space) in 2 mm steps. Notably, only those y-values were presented in which more than two thresholds contributed to the final coordinate. All individual tracts are shown on two sagital slices, one horizontal slice and one coronar slice at y=10. Table 2 provides statistics on the centre of gravity analysis at the coronar level. VLPFC = ventrolateral prefrontal cortex; PMd = dorsal premotor cortex; PMv = ventral premotor cortex; SMA = supplementary motor area.

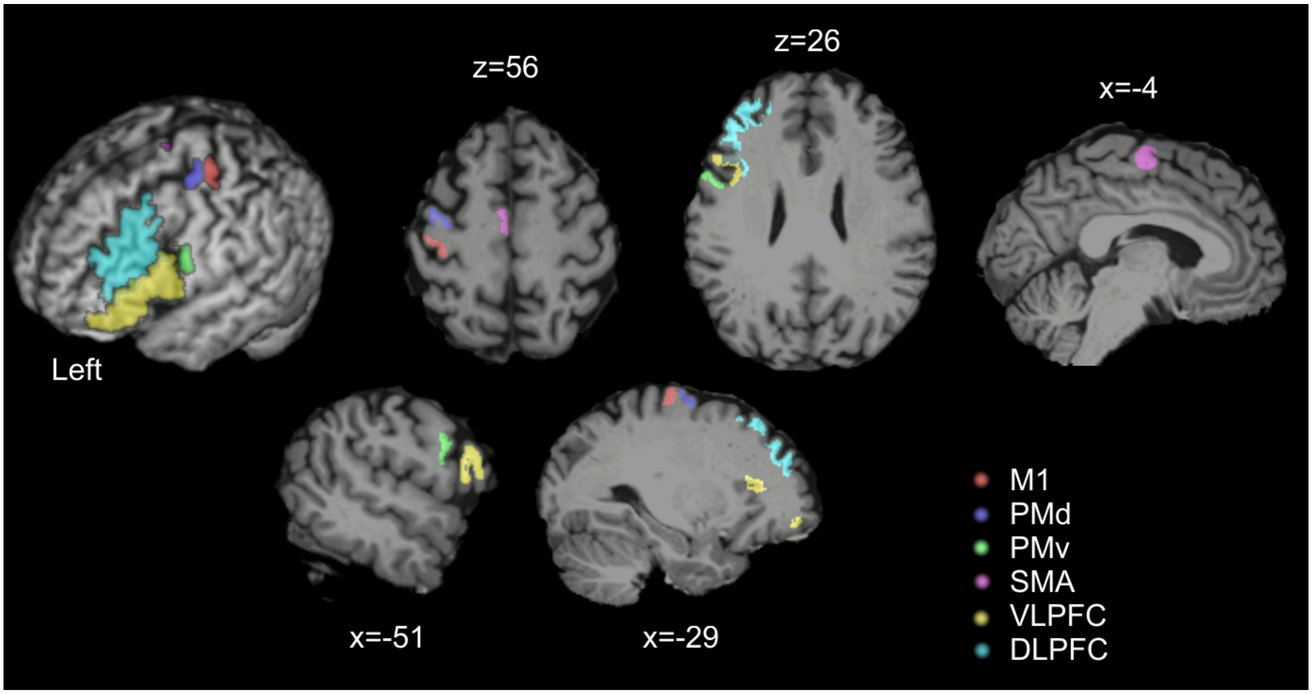

Fig. 3 | Individual example of seed/target masks plotted on an individual T1 in MNI space. x/z values are given in MNI standard space.

		DLPFC-PMd		DLPFC-PMv		DLPFC-SMA	
		AH	UH	AH	UH	AH	UH
**MD**	**Stroke**	0.00069 (±0.00006)	0.00066 (±0.00004)	0.00071 (±0.00005)	0.00071 (±0.00005)	0.00070 (±0.00005)	0.00069 (±0.00004)
	**Control**	0.00066 (±0.00004)	0.00066 (±0.00004)	0.00069 (±0.00004)	0.00069 (±0.00004)	0.00069 (±0.00005)	0.00068 (±0.00004)
**RD**	**Stroke**	0.00057 (±0.00006)	0.00054 (±0.00004)	0.00060 (±0.00005)	0.00059 (±0.00005)	0.00056 (±0.00005)	0.00056 (±0.00004)
	**Control**	0.00053 (±0.00004)	0.00053 (±0.00004)	0.00058 (±0.00005)	0.00058 (±0.00004)	0.00055 (±0.00005)	0.00054 (±0.00005)
**AD**	**Stroke**	0.00094 (±0.00006)	0.00091 (±0.00004)	0.00094 (±0.00004)	0.00094 (±0.00005)	0.00098 (±0.00005)	0.00095 (±0.00005)
	**Control**	0.00091 (±0.00004)	0.00091 (±0.00004)	0.00092 (±0.00004)	0.00092 (±0.00004)	0.00097 (±0.00005)	0.00096 (±0.00004)

		VLPFC-PMd		VLPFC-PMv		VLPFC-SMA	
		AH	UH	AH	UH	AH	UH
**MD**	**Stroke**	0.00069 (±0.00006)	0.00067 (±0.00004)	0.00075 (±0.00005)	0.00075 (±0.00004)	0.00071 (±0.00005)	0.00069 (±0.00005)
	**Control**	0.00066 (±0.00004)	0.00066 (±0.00004)	0.00075 (±0.00004)	0.00075 (±0.00004)	0.00068 (±0.00004)	0.00068 (±0.00004)
**RD**	**Stroke**	0.00057 (±0.00005)	0.00054 (±0.00004)	0.00066 (±0.00005)	0.00066 (±0.00004)	0.00057 (±0.00006)	0.00055 (±0.00005)
	**Control**	0.00053 (±0.00004)	0.00053 (±0.00004)	0.00065 (±0.00005)	0.00065 (±0.00004)	0.00054 (±0.00004)	0.00053 (±0.00004)
**AD**	**Stroke**	0.00095 (±0.00006)	0.00092 (±0.00004)	0.00095 (±0.00005)	0.00095 (±0.00004)	0.00099 (±0.00005)	0.00097 (±0.00006)
	**Control**	0.00092 (±0.00005)	0.00091 (±0.00004)	0.00094 (±0.00004)	0.00094 (±0.00004)	0.00097 (±0.00005)	0.00096 (±0.00004)

Tab. 3 | Tract-related white matter microstructure in stroke patients and healthy controls
Overview of mean MD, RD and AD values presented as means (±SD) for the affected (AH) and unaffected (UH) hemisphere. MD = mean diffusivity; RD = radial diffusivity; AD = axial diffusivity; DLPFC = dorsolateral prefrontal cortex; VLPFC = ventrolateral prefrontal cortex; PMd = dorsal premotor cortex; PMv = ventral premotor cortex; SMA = supplementary motor area.

Parameter	Side	Tract	Estimated coefficient	Confidence interval		T-Value	*P*-Value
				Lower	Upper		
**Grip**	AH	DLPFC-PMd	-111.84	-1137.50	913.82	-0.23	0.82
		DLPFC-PMv	588.94	-832.96	2010.84	0.85	0.40
		DLPFC-SMA	-68.78	-1437.47	1299.91	-0.10	0.92
	UH	DLPFC-PMd	-564.89	-2215.97	1086.18	-0.71	0.49
		DLPFC-PMv	-92.01	-1447.86	1263.84	-0.14	0.89
		DLPFC-SMA	-417.02	-1946.41	1112.37	-0.56	0.58
	AH	VLPFC-PMd	-64.27	-1147.42	1018.87	-0.12	0.90
		VLPFC-PMv	415.58	-972.96	1804.11	0.62	0.54
		VLPFC-SMA	-5.59	-1121.31	1110.13	-0.01	0.99
	UH	VLPFC-PMd	-760.70	-2411.84	890.44	-0.95	0.35
		VLPFC-PMv	1104.60	-689.85	2899.06	1.27	0.22
		VLPFC-SMA	-151.44	-1387.36	1084.48	-0.25	0.80
**Pinch**	AH	DLPFC-PMd	-636.74	-1929.14	655.67	-1.02	0.32
		DLPFC-PMv	-407.42	-2255.04	1440.21	-0.46	0.65
		DLPFC-SMA	-862.64	-2584.65	859.38	-1.03	0.31
	UH	DLPFC-PMd	-1346.95	-3415.03	721.13	-1.34	0.19
		**DLPFC-PMv**	**-2110.58**	**-3610.61**	**-610.56**	**-2.90**	**<0.01***
		DLPFC-SMA	-867.41	-2812.46	1077.64	-0.92	0.37
	AH	VLPFC-PMd	-755.49	-2111.54	600.55	-1.15	0.26
		VLPFC-PMv	443.11	-1346.39	2232.60	0.51	0.61
		VLPFC-SMA	-448.41	-1870.26	973.44	-0.65	0.52
	UH	VLPFC-PMd	-1107.45	-3218.79	1003.90	-1.08	0.29
		VLPFC-PMv	842.72	-1513.91	3199.34	0.74	0.47
		VLPFC-SMA	-277.32	-1864.00	1309.37	-0.36	0.72
**UEFM**	AH	DLPFC-PMd	7314.76	-19169.09	33798.61	0.57	0.57
		DLPFC-PMv	23941.17	-12158.23	60040.57	1.37	0.18
		DLPFC-SMA	14345.81	-20686.95	49378.56	0.85	0.41
	UH	DLPFC-PMd	-953.19	-44269.76	42363.38	-0.05	0.96
		DLPFC-PMv	-768.20	-35989.98	34453.58	-0.05	0.96
		DLPFC-SMA	-900.09	-40874.92	39074.75	-0.05	0.96
	AH	VLPFC-PMd	9290.20	-18572.17	37152.58	0.69	0.50
		VLPFC-PMv	9955.97	-26144.43	46056.38	0.57	0.57
		VLPFC-SMA	16172.82	-11987.86	44333.49	1.19	0.25
	UH	VLPFC-PMd	572.98	-43103.51	44249.48	0.03	0.98
		VLPFC-PMv	36076.65	-9601.17	81754.47	1.63	0.12
		VLPFC-SMA	152.88	-31984.32	32290.08	0.01	0.99
**MO**	AH	DLPFC-PMd	-1362.07	-10724.45	8000.31	-0.30	0.77
		DLPFC-PMv	4225.38	-8840.17	17290.92	0.67	0.51
		DLPFC-SMA	-915.77	-13416.49	11584.96	-0.15	0.88
	UH	DLPFC-PMd	-6400.53	-21399.61	8598.55	-0.88	0.39
		DLPFC-PMv	-7131.52	-19153.41	4890.38	-1.22	0.23
		DLPFC-SMA	-4350.51	-18294.45	9593.43	-0.64	0.53
	AH	VLPFC-PMd	-1287.19	-11170.61	8596.24	-0.27	0.79
		VLPFC-PMv	4301.79	-8354.77	16958.34	0.70	0.49
		VLPFC-SMA	883.93	-9302.12	11069.97	0.18	0.86
	UH	VLPFC-PMd	-6110.67	-21259.22	9037.88	-0.83	0.41
		VLPFC-PMv	11753.91	-4441.79	27949.62	1.50	0.15
		VLPFC-SMA	-1391.29	-12682.06	9899.48	-0.25	0.80

Tab. 4 | Tract-related mean MD and residual motor output after stroke

Individual multiple linear regression models were estimated for each tract to test the relationship between tract-related mean MD values and the 4 parameter grip force (Grip), pinch force (Pinch) values, UEFM score and the composite score MO. Estimated coefficients of the tract-related MD values were adjusted for age, lesioned hemisphere (dominant or non-dominant), time after stroke and CST integrity and are given with 95% confidence intervals. *P*-values are derived from each model separately and were not corrected for multiple testing. Note that after correction for 48 models (13), none of the tracts reached the level of significance. Estimated coefficients of the 4 covariates are not shown for the sake of clarity. AH = affected hemisphere, UH = unaffected hemisphere. Asterisks indicate significant tracts.

Parameter	Side	Tract	Estimated coefficient	Confidence interval		T-Value	*P*-Value
				Lower	Upper		
**Grip**	AH	DLPFC-PMd	-126.15	-1099.48	847.18	-0.27	0.79
		DLPFC-PMv	607.83	-694.68	1910.34	0.96	0.35
		DLPFC-SMA	-46.08	-1316.93	1224.76	-0.07	0.94
	UH	DLPFC-PMd	-547.76	-2144.09	1048.56	-0.71	0.49
		DLPFC-PMv	-265.60	-1480.84	949.64	-0.45	0.66
		DLPFC-SMA	-469.56	-1936.04	996.92	-0.66	0.52
	AH	VLPFC-PMd	-60.64	-1142.41	1021.14	-0.12	0.91
		VLPFC-PMv	456.54	-957.78	1870.86	0.67	0.51
		VLPFC-SMA	-15.98	-1080.21	1048.25	-0.03	0.98
	UH	VLPFC-PMd	-770.66	-2343.70	802.39	-1.01	0.32
		VLPFC-PMv	840.84	-924.54	2606.23	0.98	0.34
		VLPFC-SMA	-198.92	-1405.30	1007.45	-0.34	0.74
**Pinch**	AH	DLPFC-PMd	-667.33	-1888.54	553.88	-1.13	0.27
		DLPFC-PMv	-384.54	-2083.36	1314.28	-0.47	0.64
		DLPFC-SMA	-788.23	-2388.09	811.62	-1.02	0.32
	UH	DLPFC-PMd	-1155.17	-3170.78	860.44	-1.18	0.25
		**DLPFC-PMv**	**-2126.71**	**-3414.60**	**-838.82**	**-3.41**	**<0.01****
		DLPFC-SMA	-956.25	-2815.45	902.95	-1.06	0.30
	AH	VLPFC-PMd	-897.32	-2236.06	441.42	-1.38	0.18
		VLPFC-PMv	343.10	-1486.15	2172.35	0.39	0.70
		VLPFC-SMA	-512.95	-1863.95	838.06	-0.78	0.44
	UH	VLPFC-PMd	-1114.60	-3125.39	896.20	-1.14	0.26
		VLPFC-PMv	867.71	-1418.02	3153.44	0.78	0.44
		VLPFC-SMA	-375.09	-1921.66	1171.48	-0.50	0.62
**UEFM**	AH	DLPFC-PMd	7733.78	-17368.80	32836.37	0.64	0.53
		DLPFC-PMv	25997.41	-6687.55	58682.37	1.64	0.11
		DLPFC-SMA	10178.54	-22547.00	42904.07	0.64	0.53
	UH	DLPFC-PMd	2619.05	-39250.86	44488.96	0.13	0.90
		DLPFC-PMv	-5071.65	-36690.55	26547.24	-0.33	0.74
		DLPFC-SMA	-3650.05	-42045.93	34745.83	-0.20	0.85
	AH	VLPFC-PMd	10171.46	-17599.33	37942.25	0.76	0.46
		VLPFC-PMv	10583.87	-26212.28	47380.02	0.59	0.56
		VLPFC-SMA	14718.95	-12212.98	41650.89	1.13	0.27
	UH	VLPFC-PMd	2250.98	-39448.27	43950.23	0.11	0.91
		VLPFC-PMv	28010.50	-17233.49	73254.49	1.28	0.21
		VLPFC-SMA	-651.37	-32052.90	30750.16	-0.04	0.97
**MO**	AH	DLPFC-PMd	-1449.25	-10333.53	7435.03	-0.34	0.74
		DLPFC-PMv	4660.29	-7306.78	16627.36	0.80	0.43
		DLPFC-SMA	-1199.21	-12799.56	10401.13	-0.21	0.83
	UH	DLPFC-PMd	-5217.81	-19787.98	9352.35	-0.74	0.47
		DLPFC-PMv	-8413.10	-18983.68	2157.47	-1.64	0.11
		DLPFC-SMA	-5213.03	-18552.04	8125.98	-0.81	0.43
	AH	VLPFC-PMd	-1597.64	-11460.16	8264.88	-0.33	0.74
		VLPFC-PMv	4219.04	-8698.92	17137.01	0.67	0.51
		VLPFC-SMA	433.46	-9287.45	10154.37	0.09	0.93
	UH	VLPFC-PMd	-5927.04	-20386.72	8532.64	-0.85	0.41
		VLPFC-PMv	9743.95	-6185.04	25672.94	1.26	0.22
		VLPFC-SMA	-1984.45	-13000.33	9031.43	-0.37	0.71

Tab. 5 | Tract-related mean RD and residual motor output after stroke

Individual multiple linear regression models were estimated for each tract to test the relationship between tract-related mean RD values and the 4 behavioural parameter grip force (Grip), pinch force (Pinch) values, UEFM score and the composite score MO. Estimated coefficients of the tract-related RD values were adjusted for age, lesioned hemisphere (dominant or non-dominant), time after stroke and CST integrity and are given with 95% confidence intervals. *P*-values are derived from each model separately and were not corrected for multiple testing. Note that after correction for 48 models (13), none of the tracts reached the level of significance. Estimated coefficients of the 4 covariates are not shown for the sake of clarity. AH = affected hemisphere, UH = unaffected hemisphere. Asterisks indicate significant tracts.

Parameter	Side	Tract	Estimated coefficient	Confidence interval		T-Value	*P*-Value
				Lower	Upper		
**Grip**	AH	DLPFC-PMd	-74.28	-1138.01	989.45	-0.14	0.89
		DLPFC-PMv	369.64	-1141.20	1880.47	0.50	0.62
		DLPFC-SMA	-69.03	-1290.52	1152.46	-0.12	0.91
	UH	DLPFC-PMd	-423.21	-1914.67	1068.25	-0.59	0.56
		DLPFC-PMv	410.37	-1048.13	1868.87	0.58	0.57
		DLPFC-SMA	-166.41	-1465.67	1132.86	-0.26	0.79
	AH	VLPFC-PMd	-69.54	-1101.72	962.65	-0.14	0.89
		VLPFC-PMv	275.21	-951.03	1501.45	0.46	0.65
		VLPFC-SMA	7.10	-1083.47	1097.68	0.01	0.99
	UH	VLPFC-PMd	-509.27	-2014.42	995.88	-0.70	0.49
		VLPFC-PMv	1425.91	-254.01	3105.83	1.75	0.09
		VLPFC-SMA	-21.19	-1111.22	1068.84	-0.04	0.97
**Pinch**	AH	DLPFC-PMd	-491.68	-1844.01	860.64	-0.75	0.46
		DLPFC-PMv	-313.88	-2261.97	1634.22	-0.33	0.74
		DLPFC-SMA	-610.60	-2160.16	938.95	-0.81	0.42
	UH	DLPFC-PMd	-1287.91	-3141.15	565.34	-1.43	0.16
		DLPFC-PMv	-1280.45	-3089.89	528.99	-1.46	0.16
		DLPFC-SMA	-393.64	-2058.13	1270.85	-0.49	0.63
	AH	VLPFC-PMd	-432.53	-1747.45	882.40	-0.68	0.50
		VLPFC-PMv	488.43	-1081.59	2058.46	0.64	0.53
		VLPFC-SMA	-197.95	-1597.49	1201.59	-0.29	0.77
	UH	VLPFC-PMd	-766.01	-2693.72	1161.70	-0.82	0.42
		VLPFC-PMv	663.79	-1612.68	2940.27	0.60	0.55
		VLPFC-SMA	-23.46	-1424.80	1377.87	-0.03	0.97
**UEFM**	AH	DLPFC-PMd	4715.69	-22847.80	32279.18	0.35	0.73
		DLPFC-PMv	11375.29	-27773.81	50524.40	0.60	0.55
		DLPFC-SMA	15674.57	-15359.39	46708.53	1.04	0.31
	UH	DLPFC-PMd	-6771.73	-45673.46	32130.00	-0.36	0.72
		DLPFC-PMv	11754.54	-26062.30	49571.38	0.64	0.53
		DLPFC-SMA	3405.42	-30352.63	37163.47	0.21	0.84
	AH	VLPFC-PMd	6481.93	-20193.36	33157.23	0.50	0.62
		VLPFC-PMv	7543.05	-24283.82	39369.91	0.49	0.63
		VLPFC-SMA	15733.57	-11800.13	43267.26	1.18	0.25
	UH	VLPFC-PMd	-2676.72	-42141.53	36788.09	-0.14	0.89
		**VLPFC-PMv**	**45726.32**	**3591.77**	**87860.87**	**2.24**	**0.03***
		VLPFC-SMA	1626.14	-26672.66	29924.94	0.12	0.91
**MO**	AH	DLPFC-PMd	-1143.39	-10853.51	8566.73	-0.24	0.81
		DLPFC-PMv	1942.23	-11909.19	15793.64	0.29	0.77
		DLPFC-SMA	74.20	-11088.01	11236.41	0.01	0.99
	UH	DLPFC-PMd	-6551.19	-19993.25	6890.88	-1.01	0.32
		DLPFC-PMv	-926.74	-14338.70	12485.22	-0.14	0.89
		DLPFC-SMA	-1345.25	-13218.62	10528.12	-0.23	0.82
	AH	VLPFC-PMd	-684.90	-10113.97	8744.17	-0.15	0.88
		VLPFC-PMv	3604.14	-7545.39	14753.67	0.67	0.51
		VLPFC-SMA	1660.18	-8278.40	11598.76	0.34	0.73
	UH	VLPFC-PMd	-4609.23	-18362.31	9143.85	-0.69	0.50
		VLPFC-PMv	13705.90	-1535.86	28947.67	1.86	0.08
		VLPFC-SMA	84.84	-9873.60	10043.29	0.02	0.99

Tab. 6 | Tract-related mean AD and residual motor output after stroke

Individual multiple linear regression models were estimated for each tract to test the relationship between tract-related mean AD values and the 4 parameter grip force (Grip), pinch force (Pinch) values, UEFM score and the composite score MO. Estimated coefficients of the tract-related AD values were adjusted for age, lesioned hemisphere (dominant or non-dominant), time after stroke and CST integrity and are given with 95% confidence intervals. *P*-values are derived from each model separately and were not corrected for multiple testing. Note that after correction for 48 models (13), none of the tracts reached the level of significance. Estimated coefficients of the 4 covariates are not shown for the sake of clarity. AH = affected hemisphere, UH = unaffected hemisphere. Asterisks indicate significant tracts.

Parameter	Side	Tract	Estimated coefficient	Confidence interval		T-Value	*P*-Value
				Lower	Upper		
**Grip**	AH	DLPFC-PMd	0.15	-1.80	2.10	0.16	0.87
		DLPFC-PMv	-1.24	-3.19	0.07	-1.32	0.19
		DLPFC-SMA	-0.06	-1.76	1.64	-0.07	0.94
	UH	DLPFC-PMd	0.72	-1.71	3.14	0.61	0.55
		DLPFC-PMv	0.68	-1.26	2.62	0.73	0.47
		DLPFC-SMA	0.46	-1.60	0.07	0.46	0.65
	AH	VLPFC-PMd	-0.13	-2.24	1.98	-0.13	0.90
		VLPFC-PMv	-0.10	-2.70	2.50	-0.07	0.94
		VLPFC-SMA	-0.22	-2.05	1.61	-0.25	0.80
	UH	VLPFC-PMd	0.99	-1.51	3.49	0.82	0.42
		VLPFC-PMv	1.02	-2.53	4.57	0.59	0.56
		VLPFC-SMA	-0.01	-2.01	2.00	-0.01	0.99
**Pinch**	AH	DLPFC-PMd	1.96	-0.43	4.36	1.70	0.10
		DLPFC-PMv	0.75	-1.86	3.37	0.60	0.60
		DLPFC-SMA	2.31	-1.57	2.83	0.59	0.56
	UH	DLPFC-PMd	0.77	-2.40	3.94	0.50	0.62
		DLPFC-PMv	3.49	-0.09	0.13	3.50	<0.01**
		DLPFC-SMA	0.81	-1.85	3.48	0.63	0.53
	AH	VLPFC-PMd	2.46	-0.08	5.00	2.01	0.06
		VLPFC-PMv	0.58	-2.77	3.93	0.36	0.72
		VLPFC-SMA	0.99	-1.35	3.34	0.88	0.39
	UH	VLPFC-PMd	1.31	-1.94	4.56	0.84	0.41
		VLPFC-PMv	-0.79	-5.44	3.86	-0.35	0.73
		VLPFC-SMA	0.78	-1.81	3.38	0.63	0.54
**UEFM**	AH	DLPFC-PMd	-11.72	-62.12	38.69	-0.48	0.64
		DLPFC-PMv	-45.49	-94.18	3.19	-1.93	0.07
		DLPFC-SMA	-1.85	-46.02	42.32	-0.09	0.93
	UH	DLPFC-PMd	-11.62	-74.97	51.73	-0.38	0.71
		DLPFC-PMv	18.43	-31.96	68.82	0.76	0.46
		DLPFC-SMA	7.02	-46.52	60.56	0.27	0.79
	AH	VLPFC-PMd	-19.47	-73.65	34.72	-0.74	0.46
		VLPFC-PMv	-9.53	-76.38	57.33	-0.30	0.77
		VLPFC-SMA	-16.88	-63.90	30.14	-0.74	0.47
	UH	VLPFC-PMd	-11.90	-77.49	53.69	-0.38	0.71
		VLPFC-PMv	-12.51	-105.26	80.24	-0.28	0.78
		VLPFC-SMA	-8.91	-60.91	43.09	-0.35	0.73
**MO**	AH	DLPFC-PMd	5.07	-12.86	23.01	0.59	0.56
		DLPFC-PMv	-8.54	-26.89	9.82	-0.96	0.35
		DLPFC-SMA	1.50	-14.24	17.25	0.20	0.85
	UH	DLPFC-PMd	3.30	-19.32	25.92	0.30	0.77
		DLPFC-PMv	16.14	-0.67	32.96	1.99	0.06
		DLPFC-SMA	5.22	-13.77	24.22	0.57	0.58
	AH	VLPFC-PMd	4.55	-14.92	24.01	0.48	0.63
		VLPFC-PMv	0.16	-23.73	24.05	0.01	0.99
		VLPFC-SMA	-0.06	-17.03	16.91	-0.01	0.99
	UH	VLPFC-PMd	5.95	-17.38	29.28	0.53	0.60
		VLPFC-PMv	-0.73	-33.87	32.42	-0.05	0.96
		VLPFC-SMA	1.18	-17.41	19.78	0.13	0.91

Tab. 7 | Tract-related mean FA and residual motor output after stroke with stroke-lesion-size as additional covariate

Individual multiple linear regression models were estimated for each tract to test the relationship between tract-related mean FA values and the 4 parameters grip force (Grip), pinch force (Pinch) values, UEFM score and the composite score MO. Estimated coefficients of the tract-related FA values were adjusted for age, lesioned hemisphere (dominant or non-dominant), time after stroke, CST integrity and lesion size and are given with 95% confidence intervals. *P*-values are derived from each model separately and were not corrected for multiple testing. Note that after correction for 48 models (13), none of the tracts reached the level of significance. Estimated coefficients of the 5 covariates are not shown for the sake of clarity. AH = affected hemisphere, UH = unaffected hemisphere. Asterisks indicate significant tracts.

Parameter	Side	Tract	Estimated coefficient	Confidence interval		T-Value	*P*-Value
				Lower	Upper		
**Grip**	AH	DLPFC-PMd	0.75	-1076,14	1077.64	0.001	1.00
		DLPFC-PMv	724.00	-7258.14	2173.81	1.03	0.31
		DLPFC-SMA	-46.82	-1429.21	1335.56	-0.07	0.94
	UH	DLPFC-PMd	-638.90	-2308.19	1030.40	-0.79	0.44
		DLPFC-PMv	-148.30	-1522.19	1225.89	-0.22	0.83
		DLPFC-SMA	-460.90	-2005.16	1083.32	-0.62	0.54
	AH	VLPFC-PMd	101.48	-1065.69	1268.65	0.18	0.86
		VLPFC-PMv	655.20	-8043.92	2114.79	0.93	0.36
		VLPFC-SMA	172.30	-1027.14	1371.74	0.30	0.77
	UH	VLPFC-PMd	-823.00	-2488.74	842.83	-1.02	0.32
		VLPFC-PMv	1157.00	-649.38	2964.30	1.33	0.20
		VLPFC-SMA	-150.30	-1397.39	1096.86	-0.82	0.42
**Pinch**	AH	DLPFC-PMd	-729.90	-2097.4	637.54	-1.10	0.28
		DLPFC-PMv	-461.60	-2383.85	1460.63	-0.50	0.62
		DLPFC-SMA	-871.70	-2633.51	8902.02	-1.023	0.317
	UH	DLPFC-PMd	-1337.00	-3465.01	790.67	-1.30	0.21
		DLPFC-PMv	-2113.00	-3656.60	-569.39	-2.83	<0.01**
		DLPFC-SMA	-856.90	-2851.76	1138.02	-0.89	0.38
	AH	VLPFC-PMd	-922.60	-2391.65	54643.54	-1.30	0.21
		VLPFC-PMv	423.7	-1504.83	2352.28	0.46	0.65
		VLPFC-SMA	-572.70	-2118.99	973.67	-0.77	0.45
	UH	VLPFC-PMd	-1096.00	-3264.03	1073.88	-1.05	0.31
		VLPFC-PMv	829.60	-1586.73	3245.85	0.71	0.49
		VLPFC-SMA	-277.70	-1900.57	1345.13	-0.35	0.73
**UEFM**	AH	DLPFC-PMd	4784.00	-23114.31	32683.07	0.36	0.73
		DLPFC-PMv	21920.00	-15433.06	59264.46	1.21	0.24
		DLPFC-SMA	13800.00	-21615.25	49220.76	0.81	0.43
	UH	DLPFC-PMd	786.50	-43163.31	44736.39	0.04	0.97
		DLPFC-PMv	643.90	-35092.12	36379.92	0.04	0.97
		DLPFC-SMA	172.70	-40273.96	40619.30	0.01	0.99
	AH	VLPFC-PMd	6119.00	-24107.92	3645.04	0.42	0.68
		VLPFC-PMv	6039.00	-32492.92	44570.40	0.32	0.75
		VLPFC-SMA	13840.00	-16797.59	44486.05	0.94	0.36
	UH	VLPFC-PMd	2036.00	-42199.50	46272.24	0.10	0.92
		VLPFC-PMv	34990.00	-11308.24	81288.28	1.56	0.13
		VLPFC-SMA	122.80	-32318.11	32563.72	0.01	0.99
**MO**	AH	DLPFC-PMd	-1625.00	-11580.02	8329.59	-0.34	0.74
		DLPFC-PMv	4238.00	-9383.72	17859.12	0.64	0.53
		DLPFC-SMA	-945.14	-13750.54	11860.26	-0.15	0.88
	UH	DLPFC-PMd	-6380.00	-21815.14	9055.35	-0.86	0.40
		DLPFC-PMv	-7134.00	-19505.80	5237.10	-1.19	0.25
		DLPFC-SMA	-4317.00	-18625.19	9990.44	-0.62	0.54
	AH	VLPFC-PMd	-1690.00	-12489.13	9109.08	-0.32	0.75
		VLPFC-PMv	4520.90	-9117.11	18158.91	0.69	0.50
		VLPFC-SMA	781.25	-10350.12	11912.61	0.15	0.89
	UH	VLPFC-PMd	-6082.00	-21645.53	9482.06	-0.81	0.43
		VLPFC-PMv	11740.00	-4874.57	28358.59	1.46	0.16
		VLPFC-SMA	-1393.00	-12949.80	10164.24	-0.25	0.81

Tab. 8 | Tract-related mean MD and residual motor output after stroke with stroke-lesion-size as additional covariate

Individual multiple linear regression models were estimated for each tract to test the relationship between tract-related mean MD values and the 4 parameter grip force (Grip), pinch force (Pinch) values, UEFM score and the composite score MO. Estimated coefficients of the tract-related MD values were adjusted for age, lesioned hemisphere (dominant or non-dominant), time after stroke, CST integrity and lesion size and are given with 95% confidence intervals. *P*-values are derived from each model separately and were not corrected for multiple testing. Note that after correction for 48 models (13), none of the tracts reached the level of significance. Estimated coefficients of the 5 covariates are not shown for the sake of clarity. AH = affected hemisphere, UH = unaffected hemisphere. Asterisks indicate significant tracts.

Parameter	Side	Tract	Estimated coefficient	Confidence interval		T-Value	*P*-Value
				Lower	Upper		
**Grip**	AH	DLPFC-PMd	11.83	-1084.43	1107.20	0.02	0.98
		DLPFC-PMv	414.6	-1111.39	1940.58	0.56	0.58
		DLPFC-SMA	-111.10	-1347.43	1125.32	-0.19	0.85
	UH	DLPFC-PMd	-484.60	-1992.68	1023.55	-0.67	0.51
		DLPFC-PMv	315.50	-1184.33	1815.32	0.44	0.67
		DLPFC-SMA	-235.60	-1555.05	1083.90	-0.37	0.72
	AH	VLPFC-PMd	65.52	-1030.17	1161.21	0.12	0.90
		VLPFC-PMv	450.50	-830.25	1731.23	0.73	0.47
		VLPFC-SMA	155.79	-998.17	1309.75	0.28	0.78
	UH	VLPFC-PMd	-553.30	-2072.36	9657.98	-0.75	0.46
		VLPFC-PMv	1412.00	-282.03	3106.85	1.72	0.10
		VLPFC-SMA	-17.28	-1117.27	1082.71	-0.03	0.97
**Pinch**	AH	DLPFC-PMd	-542.70	-1951.28	865.92	-0.80	0.43
		DLPFC-PMv	-330.30	-2327.32	1666.64	-0.34	0.74
		DLPFC-SMA	-600.60	-2191.91	990.74	-0.78	0.44
	UH	DLPFC-PMd	-1280.00	-3185.23	626.00	-1.39	0.18
		DLPFC-PMv	-1285.00	-3167.26	597.78	-1.41	0.98
		DLPFC-SMA	-376.90	-2093.68	1339.84	-0.45	0.65
	AH	VLPFC-PMd	-527.80	-1937.79	882.13	-0.77	0.45
		VLPFC-PMv	478.20	-1197.13	2153.51	0.59	0.56
		VLPFC-SMA	-269.50	-1771.27	1232.19	-0.37	0.71
	UH	VLPFC-PMd	-755.20	-2732.23	1221.92	-0.79	0.44
		VLPFC-PMv	668.90	-1659.62	2997.43	0.59	0.56
		VLPFC-SMA	-24.82	-1458.16	1408.52	-0.04	0.97
**UEFM**	AH	DLPFC-PMd	2635.00	-25809.26	31078.95	0.19	0.85
		DLPFC-PMv	10330.00	-29337.55	49989.21	0.54	0.56
		DLPFC-SMA	16850.00	-14458.37	48160.42	1.11	0.28
	UH	DLPFC-PMd	-5357.00	-44840.86	34127,27	-0.28	0.78
		DLPFC-PMv	14910.00	-23676.03	53502.76	0.80	0.43
		DLPFC-SMA	5166.00	-29140.40	39471.56	0.31	0.76
	AH	VLPFC-PMd	3519.00	-24913.73	31951.09	0.26	0.80
		VLPFC-PMv	4307.00	-29292.46	37907.24	0.27	0.79
		VLPFC-SMA	13560.00	-15888.32	43016.40	0.95	0.35
	UH	VLPFC-PMd	-1621.00	-41560.64	38319.08	-0.08	0.93
		VLPFC-PMv	46100.00	3765.87	88442.39	2.25	<0.05*
		VLPFC-SMA	1.10	-27042.15	30093.99	0.81	0.42
**MO**	AH	DLPFC-PMd	-1303.00	-11442.19	8836.54	-0.27	0.79
		DLPFC-PMv	1897.36	-12317.10	16111.82	0.28	0.78
		DLPFC-SMA	127.22	-11338.64	11593.09	0.02	0.98
	UH	DLPFC-PMd	-6537.00	-20361.98	7287.09	-0.98	0.34
		DLPFC-PMv	-820.10	-14771.52	13131.32	-0.12	0.90
		DLPFC-SMA	-1283.00	-13533.82	10968.64	0.09	0.93
	AH	VLPFC-PMd	-937.92	-11086.96	9211.12	-0.19	0.85
		VLPFC-PMv	3723.51	-8172.91	15619.92	0.65	0.52
		VLPFC-SMA	1645.00	-9043.68	12332.92	0.32	0.75
	UH	VLPFC-PMd	-4578.00	-18690.29	9534.51	-0.67	0.51
		VLPFC-PMv	13730.00	-1871.32	29329.16	1.82	0.08
		VLPFC-SMA	79.90	-10113.80	10273.60	0.02	0.99

Tab. 9 | Tract-related mean AD and residual motor output after stroke with stroke-lesion-size as additional covariate

Individual multiple linear regression models were estimated for each tract to test the relationship between tract-related mean AD values and the 4 parameter grip force (Grip), pinch force (Pinch) values, UEFM score and the composite score MO. Estimated coefficients of the tract-related AD values were adjusted for age, lesioned hemisphere (dominant or non-dominant), time after stroke, CST integrity and lesion size and are given with 95% confidence intervals. *P*-values are derived from each model separately and were not corrected for multiple testing. Note that after correction for 48 models (13), none of the tracts reached the level of significance. Estimated coefficients of the 5 covariates are not shown for the sake of clarity. AH = affected hemisphere, UH = unaffected hemisphere. Asterisks indicate significant tracts.

Parameter	Side	Tract	Estimated coefficient	Confidence interval		T-Value	*P*-Value
				Lower	Upper		
**Grip**	AH	DLPFC-PMd	-10.41	-1040.78	1019.95	-0.02	0.98
		DLPFC-PMv	771.90	-563.83	2108.61	1.20	0.24
		DLPFC-SMA	6.64	-1282.75	1296.04	0.01	0.99
	UH	DLPFC-PMd	-617.40	-2231.03	996.26	-0.79	0.44
		DLPFC-PMv	-295.50	-1522.47	931.53	-0.50	0.62
		DLPFC-SMA	-485.10	-1964.03	993.89	-0.68	0.50
	AH	VLPFC-PMd	111.36	-1058.98	1281.71	0.20	0.41
		VLPFC-PMv	706.20	-779.86	2192.35	0.98	0.34
		VLPFC-SMA	155.39	-991.34	1302.11	0.28	0.78
	UH	VLPFC-PMd	-829.80	-2416.25	756.74	-1.08	0.29
		VLPFC-PMv	922.30	-859.86	2704.53	1.07	0.30
		VLPFC-SMA	-199.50	-1416.69	1017.76	-0.34	0.74
**Pinch**	AH	DLPFC-PMd	-776.50	-2076.70	523.67	-1.24	0.23
		DLPFC-PMv	-451.00	-2234.24	1332.16	-0.52	0.61
		DLPFC-SMA	-815.10	-2458.06	827.87	-1.03	0.32
	UH	DLPFC-PMd	-1144.00	-3217.38	929.97	-1.14	0.27
		DLPFC-PMv	-2124.00	-3444.86	-803.31	-3.33	<0.01**
		DLPFC-SMA	-951.60	-2854.21	950.96	-1.04	0.31
	AH	VLPFC-PMd	-1095.00	-2546.58	356.13	-1.56	0.13
		VLPFC-PMv	311.60	-1660.61	2283.73	0.33	0.75
		VLPFC-SMA	-646.90	-2117.44	823.688	-0.91	0.37
	UH	VLPFC-PMd	-1104.00	-3168.77	961.39	-1.11	0.28
		VLPFC-PMv	851.20	-1499.75	3202.18	0.75	0.46
		VLPFC-SMA	-374.90	-1956.73	1206.93	-0.49	0.83
**UEFM**	AH	DLPFC-PMd	5203.00	-21469.08	31875.61	0.40	0.69
		DLPFC-PMv	24130.00	-10087.79	58338.17	1.46	0.16
		DLPFC-SMA	8923.00	-24350.72	42196.60	0.56	0.58
	UH	DLPFC-PMd	4293.00	-38151.85	46738.51	0.21	0.84
		DLPFC-PMv	-4347.00	-36337.80	27644.15	-0.28	0.78
		DLPFC-SMA	1.10	-42039.31	35514.33	0.81	0.86
	AH	VLPFC-PMd	7059.00	-23216.19	37334.76	0.48	0.63
		VLPFC-PMv	6617.00	-32687.23	45921.77	0.35	0.73
		VLPFC-SMA	12390.00	-16971.84	41743.80	0.87	0.39
	UH	VLPFC-PMd	3646.00	-38573.77	45865.63	0.18	0.86
		VLPFC-PMv	26420.00	-19627.06	72467.56	1.19	0.25
		VLPFC-SMA	-637.60	-32335.81	31060.64	-0.04	0.97
**MO**	AH	DLPFC-PMd	-1752.00	-11270.55	7766.38	-0.38	0.83
		DLPFC-PMv	4757.76	-7832.91	17348.43	0.78	0.44
		DLPFC-SMA	-1279.00	-13215.44	10656.70	-0.22	0.83
	UH	DLPFC-PMd	-5187.00	-20177.12	9802.37	-0.72	0.48
		DLPFC-PMv	-8406.00	-19247.18	2435.74	-1.60	0.12
		DLPFC-SMA	-5197.00	-18856.39	8461.96	-0.79	0.44
	AH	VLPFC-PMd	-2068.00	-12885.43	8750.42	-0.40	0.70
		VLPFC-PMv	4427.82	-9499.81	18355.44	0.67	0.52
		VLPFC-SMA	276.39	-10367.73	10920.51	0.05	0.96
	UH	VLPFC-PMd	-5900.00	-20754.93	8955.10	-0.82	0.42
		VLPFC-PMv	9750.00	-6641.21	26140.93	1.23	0.23
		VLPFC-SMA	540.60	-13259.45	9291.90	0.11	0.91

Tab. 10 | Tract-related mean RD and residual motor output after stroke with stroke-lesion-size as additional covariate

Individual multiple linear regression models were estimated for each tract to test the relationship between tract-related mean RD values and the 4 parameter grip force (Grip), pinch force (Pinch) values, UEFM score and the composite score MO. Estimated coefficients of the tract-related RD values were adjusted for age, lesioned hemisphere (dominant or non-dominant), time after stroke, CST integrity and lesion size and are given with 95% confidence intervals. *P*-values are derived from each model separately and were not corrected for multiple testing. Note that after correction for 48 models (13), none of the tracts reached the level of significance. Estimated coefficients of the 5 covariates are not shown for the sake of clarity. AH = affected hemisphere, UH = unaffected hemisphere. Asterisks indicate significant tracts.

3 References

1. Fischl B, Salat DH, Busa E, Albert M, Dieterich M, Haselgrove C, van der Kouwe A, Killiany R, Kennedy D, Klaveness S, et al. Whole Brain Segmentation. *Neuron* (2002) **33**:341–355. doi:10.1016/S0896-6273(02)00569-X
2. Geyer S, Ledberg A, Schleicher A, Kinomura S, Schormann T, Bürgel U, Klingberg T, Larsson J, Zilles K, Roland PE. Two different areas within the primary motor cortex of man. *Nature* (1996) **382**:805–7. doi:10.1038/382805a0
3. Nachev P, Kennard C, Husain M. Functional role of the supplementary and pre-supplementary motor areas. *Nat Rev Neurosci* (2008) **9**:856–69. doi:10.1038/nrn2478
4. Tomassini V, Jbabdi S, Klein JC, Behrens TEJ, Pozzilli C, Matthews PM, Rushworth MFS, Johansen-Berg H. Diffusion-weighted imaging tractography-based parcellation of the human lateral premotor cortex identifies dorsal and ventral subregions with anatomical and functional specializations. *J Neurosci* (2007) **27**:10259–69. doi:10.1523/JNEUROSCI.2144-07.2007
5. Mayka MA, Corcos DM, Leurgans SE, Vaillancourt DE. Three-dimensional locations and boundaries of motor and premotor cortices as defined by functional brain imaging: a meta-analysis. *Neuroimage* (2006) **31**:1453–74. doi:10.1016/j.neuroimage.2006.02.004
6. Eickhoff SB, Stephan KE, Mohlberg H, Grefkes C, Fink GR, Amunts K, Zilles K. A new SPM toolbox for combining probabilistic cytoarchitectonic maps and functional imaging data. *Neuroimage* (2005) **25**:1325–35. doi:10.1016/j.neuroimage.2004.12.034
7. Rushworth MF, M Owen A. The functional organization of the lateral frontal cortex: conjecture or conjuncture in the electrophysiology literature? *Trends Cogn Sci* (1998) **2**:46–53.
8. Schulz R, Wessel MJ, Zimerman M, Timmermann JE, Gerloff C, Hummel FC. White Matter Integrity of Specific Dentato-Thalamo-Cortical Pathways is Associated with Learning Gains in Precise Movement Timing. *Cereb Cortex* (2015) **25**:1707–1714. doi:10.1093/cercor/bht356
9. Schulz R, Frey BM, Koch P, Zimerman M, Bönstrup M, Feldheim J, Timmermann JE, Schön G, Cheng B, Thomalla G, et al. Cortico-Cerebellar Structural Connectivity Is Related to Residual Motor Output in Chronic Stroke. *Cereb Cortex* (2017) **27**:635–645. doi:10.1093/cercor/bhv251
10. Hughes EJ, Bond J, Svrckova P, Makropoulos A, Ball G, Sharp DJ, Edwards AD, Hajnal J V, Counsell SJ. Regional changes in thalamic shape and volume with increasing age. *Neuroimage* (2012) **63**:1134–42. doi:10.1016/j.neuroimage.2012.07.043
11. Schulz R, Koch P, Zimerman M, Wessel M, Bönstrup M, Thomalla G, Cheng B, Gerloff C, Hummel FC. Parietofrontal motor pathways and their association with motor function after stroke. *Brain* (2015) **138**:1949–1960. doi:10.1093/brain/awv100
12. Schulz R, Braass H, Liuzzi G, Hoerniss V, Lechner P, Gerloff C, Hummel FC. White matter integrity of premotor-motor connections is associated with motor output in chronic stroke patients. *NeuroImage Clin* (2015) **7**:82–6. doi:10.1016/j.nicl.2014.11.006
13. Benjamini Y, Yekutieli D. The control of the false discovery rate in multiple testing under dependency. *Ann Stat* (2001) **29**:1165–1188.
